# Development of High Flux Nanocomposite Polyphenylsulfone/Oxidized Multiwalled Carbon Nanotubes Membranes for Ultrafiltration Using the Systems with Critical Solution Temperatures

**DOI:** 10.3390/membranes12080724

**Published:** 2022-07-22

**Authors:** Tatiana V. Plisko, Katsiaryna S. Burts, Alexandr V. Bildyukevich

**Affiliations:** Institute of Physical Organic Chemistry, National Academy of Sciences of Belarus, 220072 Minsk, Belarus; katyaburt@gmail.com (K.S.B.); uf@ifoch.bas-net.by (A.V.B.)

**Keywords:** nanocomposite membrane, ultrafiltration, polyphenylsulfone, critical solution temperature, multiwalled carbon nanotubes

## Abstract

The study deals with the investigation of the effect of the modification of polyphenylsulfone (PPSU) flat sheet membranes for ultrafiltration using oxidized multiwalled carbon nanotubes (O-MWCNT) in order to enhance membrane permeability and antifouling performance. The effect of O-MWCNT loading to the PPSU-polyethylene glycol (PEG-20,000, M_n_ = 20,000 g·mol^−1^)-polyvinylpyrrolidone (PVP K-30, M_n_ = 40,000 g·mol^−1^)-N-methy-2-pyrrolidinone (NMP) colloid systems on the phase state and viscosity was studied. It was found that PPSU-PEG-20,000-PVP K-30-O-MWCNT-NMP colloid systems feature a gel point (T = 35–37 °C) and demixing temperature (T = 127–129 °C) at which two bulk phases are formed and a polymer system delaminates. According to the study of the phase state and viscosity of these colloid systems, a method for the preparation of high flux PPSU membranes is proposed which includes processing of the casting solution at the temperature higher than gel point (40 °C) and using a coagulation bath temperature lower than gel point (25 °C) or lower than demixing temperature (40 °C and 70 °C). Membrane structure, topology and hydrophilic-hydrophobic balance were investigated by scanning electron microscopy (SEM), atomic force microscopy (AFM) and water contact angle measurements. The effect of coagulation bath temperature and O-MWCNT concentration on the membrane separation and antifouling performance in ultrafiltration of human serum albumin and humic acids solutions was studied. It was found that the modification of PPSU ultrafiltration membranes by O-MWCNTs yielded the formation of a thinner selective layer and hydrophilization of the membrane surface (water contact angle decreased from 53–56° for the reference PPSU membrane down to 33° for the nanocomposite membrane with the addition of 0.19 wt.% O-MWCNT). These changes resulted in the increase in membrane flux (from 203–605 L·m^−2^·h^−1^ at transmembrane pressure of 0.1 MPa for the reference membrane up to 512–983 L·m^−2^·h^−1^ for nanocomposite membrane with the addition of 0.19 wt.% O-MWCNT depending on coagulation bath temperature) which significantly surpasses the performance of PPSU ultrafiltration membranes reported to date while maintaining a high level of human serum albumin rejection (83–92%). It was revealed that nanocomposite membrane demonstrated better antifouling performance (the flux recovery ratio increased from 47% for the reference PPSU membrane up to 62% for the nanocomposite membrane) and higher total organic carbon removal compared to the reference PPSU membrane in humic acids solution ultrafiltration.

## 1. Introduction

Membrane technologies are known to be effective methods of separation of different mixtures: liquids, gases, as well as dispersions. Pressure driven membrane processes, like microfiltration, ultrafiltration, nanofiltration, and reverse osmosis are widely used in industry. Ultrafiltration is one of the most applied pressure-driven membrane separation processes for the separation of oligomers or polymers from their solutions with low molecular weight substances, and the separation and concentration of colloidal particles, pharmaceutical substances and biological compounds [1].

Polyphenylsulfone (PPSU), being one of the polymers of the polysulfone group, is a chemical and physical resistant polymer with good mechanical properties, high hydrophobicity as well as relatively easy manufacturing methods [2,3]. It is known that PPSU features higher chemical resistance to alkaline cleaning and organic solvents, hydrolysis and operation temperature compared to polysulfone (PSf) and polyethersulfone (PES) [4,5,6]. These advantages result in using PPSU membranes in ultrafiltration [7,8,9], nanofiltration [10,11,12,13], forward osmosis [14], pervaporation [15,16,17], and gas separation [16,18,19], in membrane contactors [20] and proton exchange membrane fuel cells [21]. PPSU membranes were developed for water treatment [22,23,24,25], water desalination [14,26], for dye removal [3,21,27,28,29], oil in water separation [30,31,32], water purification from natural organic matter (NOM) [33], removal of heavy metal ions [10,34], CO_2_/CH_4_ gas separation [18], removal of free fatty acid from crude palm oil [35], removal of kinetic hydrate inhibitor from water [36,37], the separation of biobutanol from ABE mixtures [5] and cumene from water via pervaporation [17].

The main problems of developing PPSU ultrafiltration membranes are the low permeability and high hydrophobicity [38,39]. The low flux of PPSU membranes is due to the limitations of the variation of the casting solution composition due to the low miscibility of PPSU with commonly used pore-forming additives (PEG, polyvinypyrrolidone (PVP)) in aprotic amide solvents (N-methy-2-pyrrolidinone (NMP), N,N-dimethylformamide (DMF), N,N-dimethylacetamide (DMAc)) [6].

The hydrophobicity of PPSU (water contact angle of PPSU membrane was found to be above 73° [38]) causes the membrane fouling and flux decline which leads to the increase in the operational costs because of frequent chemical cleaning. Membrane fouling is known to occur due to the adsorption of colloids or solid particles, dissolved proteins, humic acids and other hydrophobic foulants in the feed on the membrane surface and inside membrane pores [39,40]. One of the most effective methods of decreasing membrane fouling is to enhance membrane surface hydrophilicity. In the case of PPSU membranes, surface hydrophilization was accomplished via (i) chemical modification (sulfonation or amination); (ii) blending with hydrophilic polymers (polyvinylpyrrolidone (PVP), polyethylene glycol (PEG), hyperbranched polyethylenimine (PEI)), nanoparticles (ZnO, TiO_2_, graphene oxide (GO), activated carbon (AC), etc.); and (iii) surface coating or adsorption [41].

The effect of the addition of hydrophilic water-soluble polymers and oligomers (PVP [3,34,42,43], PEG [6,43,44,45], hyperbranched PEI [46]), surfactants (Tween 80 [43]), low molecular weight substances (1,2-propylene glycol (PG) [43], glycerol [18], alcohols), inorganic nanoparticles (multiwalled carbon nanotubes (MWCNT) [34], silver-doped multi-walled carbon nanotube [47], nano tin oxide (IV) (SnO_2_) [3], nano silica (SiO_2_) [48] and fumed silica particles [5], binary zinc-magnesium oxides [49], nano zirconium oxide (ZrO_2_) [9], nano titanium oxide (TiO_2_) [2], BiOCl nanowafers loaded on activated charcoal [30], GO [37,45], AC [50]), modified nanoparticles (gum arabic-graphene [51], dendritic fibrous nanosilica (KCC-1-nPr-NH-AcCys) [24]), zeolites (ZSM-5 [31]) and metal organic frameworks (copper-1,3,5-benzenetricarboxylate (Cu-BTC) [52], and zeolitic imidazolate frameworks ZIF-8 [27]) were investigated and reported. The performance of the developed PPSU and PPSU nanocomposite ultrafiltration membranes reported to date is summarized in Table 1.

For instance, Xiao et al. introduced GO and PEG, as a pore-forming agent, into a PPSU-based dope solution for preparation of mixed matrix membranes [45]. The presence of GO and PEG enhanced the hydrophilicity, thermal stability, fouling resistance, as well as pure water flux (up to 231.7 L·m^−2^·h^−1^), which is an increase by 83% compared with the reference PPSU membrane. The rejection of the bovine serum albumin (BSA) ranged between 90 and 98% [45].

The effect of the addition of PEG to the casting solution based on sulfonated PPSU (sPPSU) was studied with emphasis on the chemical interactions between PEG and sPPSU and the rheological properties of the dope solution [55]. The resultant membranes demonstrated enhanced pure water flux (up to 35 L·m^−2^·h^−1^), improved hydrophilicity of the surface of the selective layer (water contact angle decreased down to 53.40°) and higher mechanical strength compared to the reference membrane [55].

To improve the antifouling and separation properties of hollow fiber membranes in protein solution ultrafiltration, nano TiO_2_ was introduced to the casting solution based on PPSU and sPPSU [2]. It was found that membrane modification using nano TiO_2_ yields 25% higher flux compared to the reference membrane and improved thermal, mechanical and antifouling properties [2].

It was found that the addition of metal-organic frameworks (MOF) of a different nature to the PPSU casting solution has a different effect on PPSU membrane performance: ZIF-8 reduced the flux [27], but Cu-BTC increased it [56]. However, the rejection was found to be higher in both cases.

MWCNT and functionalized MWCNT are perspective modifying agents that were applied for the modification of the PPSU [34], PSf [57], polyvinylidene fluoride (PVDF) [58], PES [59,60] and polyacrylonitrile membranes [61].

For instance, Arockiasamy et al. [54] found that the incorporation of MWCNT and carboxylated MWCNT into casting PPSU solution resulted in the improvement of the flux and antifouling performance of the developed membranes (Table 1). This enhancement is supposed to be due to the increase in the segmental gap between the PPSU and MWCNT during membrane formation and, in case of functionalized MWCNT, is due to the addition of hydrophilic –COOH moieties in the blend membrane [54].

In its turn, Chandrashekhar Nayak et al. [34] obtained PPSU/MWCNT/PVP/NMP nanofiltration membranes for heavy metals removal. It was found that the addition of MWCNT into the membrane matrix resulted in the increase in membrane permeability and the rejection of Pb^2+^, Hg^2+^, and Cd^2+^ ions.

However, the modification of PPSU membranes via the blending of nanoparticles to the casting solution did not allow to significantly increase membrane permeability according to the analysis of the literature (Table 1). From another point of view, the addition of hydrophilic nanoparticles to the PPSU casting solution was found to be an efficient technique for the hydrophilization of the membrane selective layer surface [2,25,30,48,51,54]. The idea of this study was to combine two approaches of PPSU membrane modification: blending the PPSU casting solution with hydrophilic carboxylated MWCNT(O-MWCNT) and the application of the solutions with critical solution temperatures to obtain hydrophilic high flux PPSU membranes. The successful development of high flux PPSU ultrafiltration membranes using the systems with critical solutions temperatures was carried out by our research group before and reported in [6,44]. To the best of our knowledge, the combination of these two approaches was applied for the first time for the development of PPSU ultrafiltration membranes.

Polymer systems with critical solution temperatures are perspective for PPSU and PSf membrane preparation due to the possibility to obtaining highly permeable porous membrane structures due to the combination of non-solvent induced phase separation (NIPS) and temperature induced phase separation (TIPS) [6,44,62]. In our previous works [6,44], the phase state and viscosity of PPSU-PEG-NMP systems with different molecular weights of PEG featuring the upper critical solution temperature, lower critical solution temperature and gel point were investigated. It was found that the one-phase solution region narrowed with the increase in PEG molecular weight because of the decline in compatibility between PPSU and PEG. Based on this research, the novel method for preparation of high flux PPSU ultrafiltration membranes was proposed and the optimal casting solution composition was determined in this study. For the first time, a new casting solution composition was proposed which contains PPSU, PEG (M_w_ = 20,000 g·mol^−1^), PVP (Mw = 40,000 g·mol^−1^), and carboxylated MWCNT in NMP.

In this work, novel PPSU-PEG-PVP-oxidized multiwalled carbon nanotubes (O-MWCNT) membranes with enhanced flux and antifouling performance were developed using the systems with critical solution temperatures. For the first time the phase state of the PPSU-PEG-PVP-O-MWCNTs-NMP system as well as viscosity was investigated and the fragment of phase diagram was obtained. Based on these studies for the first time, a method for preparation of high flux nanocomposite PPSU/O-MWCNTs membranes is proposed which includes the processing of the casting solution at the temperature higher than gel point (40 °C) and using coagulation bath temperature lower than gel point (25 °C) or lower than demixing temperature (40 °C and 70 °C). The effect of the O-MWCNT content in the PPSU casting solution and the coagulation bath temperature via membrane preparation by NIPS and the combination of NIPS and TIPS on the membrane structure, topography and hydrophilic-hydrophobic properties of the selective layer surface, separation performance, rejection and antifouling stability was revealed.

## 2. Materials and Methods

### 2.1. Materials

The polymer casting solutions for membrane preparation were prepared using polyphenylsulfone (PPSU) (M_w_ = 48,000 g·mol^−1^, Ultrason P 3010 NAT, BASF, Ludwigshafen, Germany) as a membrane material, polyethylene glycol (PEG-20,000, *M_n_* = 20,000 g·mol^−1^, Fluka, Munich, Germany) as a pore-forming additive; polyvinylpyrrolidone PVP (PVP K-30, M_w_ = 40,000 g·mol^−1^, Fluka, Munich, Germany) was applied as a hydrophilizing agent as well as the dispersing agent for oxidized (carboxylated) multiwalled carbon nanotubes (O-MWCNT), and N-methyl-2-pyrrolidinone (NMP, EKOS-1, Moscow, Russia, 99% purity) served as a solvent. Human serum albumin (HSA, Sigma Aldrich, St. Louis, MO, USA) solution (0.05 wt.%) in phosphate buffer (0.05 M, pH = 7.2) was used as a testing solution for investigations of membrane separation performance in ultrafiltration.

To prepare the 0.005 wt.% humic acids (HAs) solution, Hydrohumin fertilizer (Biochem, Svisloch, Belarus, content of HAs was 50 wt.%) was dissolved in tap water. This solution was applied to evaluate the antifouling and separation performance of the reference PPSU and nanocomposite PPSU/O-MWCNT membranes.

MWCNTs (d = 20–40 nm, number of walls 7–12, purity > 90%, catalyst content 5%, amorphous carbon content < 5%) were obtained by pyrolysis of a propane–butane mixture using a copper–nickel catalyst at a temperature of 600 °C using a chemical vapor deposition method.

### 2.2. Preparation of Oxidized Multiwalled Carbon Nanotubes (O-MWCNT)

**Figure 1:**
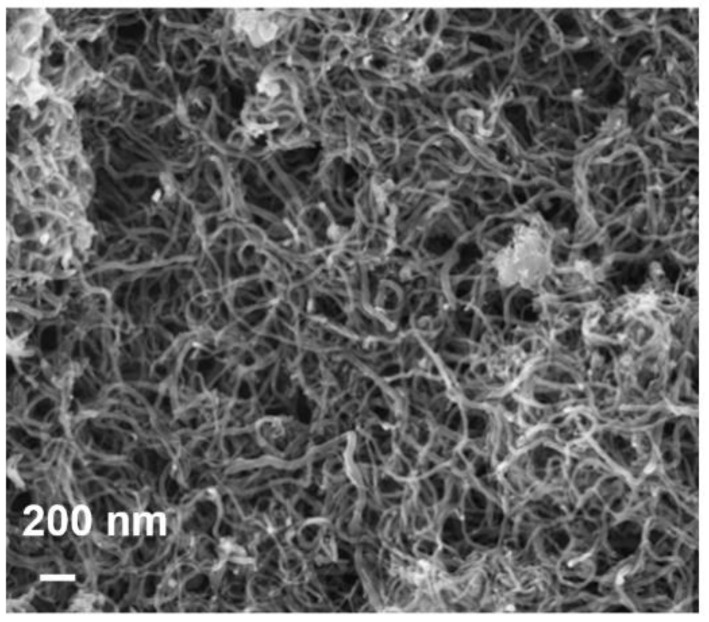
Scanning electron micrograph of O-MWCNTs.

Oxidation (carboxylation) of MWCNTs was performed with a previously prepared mixture (1:1 by weight) of concentrated sulfuric (93–98 wt.%, Merck, Rahway, NJ, USA) and nitric (70 wt.%, Merck, Rahway, NJ, USA) acids. A 50 mL round bottom vessel was loaded with 1.0 g of MWCNTs and filled with 30 mL of the mixture of the mineral acids. The resulting mixture was stirred vigorously at T = 70 °C for 24 h. The presence of oxygen-containing groups (hydroxyl, carbonyl, carboxylic, anhydride groups) was confirmed by the Fourier transform infrared spectroscopy method and reported in [63]. The product was then filtered off using a Shott filter (pore size 100 μm) and washed successively with water, ethanol, and dried in the air at ambient temperature. The images of O-MWCNT were taken using a LEO 1420 scanning electron microscope (SEM) (LEO Electron Microscopy Inc., Thornwood, NY, USA). A layer of gold on the O-MWCNT powder for SEM studies was applied by cathode sputtering in an EMITECH 550X vacuum setup (Quorum Technologies Ltd., Laughton, UK). Figure 1 demonstrates the SEM micrograph of O-MWCNTs.

### 2.3. Preparation of O-MWCNT Dispersions in NMP

Two types of dispersions of O-MWCNT in NMP were prepared: by using PVP K-30 as a dispersing agent and without it. To obtain O-MWCNT dispersions in NMP with an addition of PVP K-30 as a dispersing agent, firstly, the PVP K30 solution in NMP was obtained using the magnetic stirrer. The PVP K-30 concentration in NMP was 5; 7; 15; 20; 30; 51.4 g·L^−1^. Thereafter, 5.14 g·L^−1^ O-MWCNTs was added to NMP or NMP-PVP K-30 solution. O-MWCNTs were dispersed in NMP or NMP-PVP K-30 solution via ultrasonic treatment (180 min) in ultrasonic bath (Ultron, Poland, Olsztyn, ν = 21 kHz), followed by filtration through paper filter (Whatman, grade 1, pore size 11 μm) to remove the non-dispersed nanoparticles.

### 2.4. Characterization of O-MWCNT Dispersions

#### 2.4.1. Determination of the Concentration of Dispersed O-MWCNT

**Figure 2:**
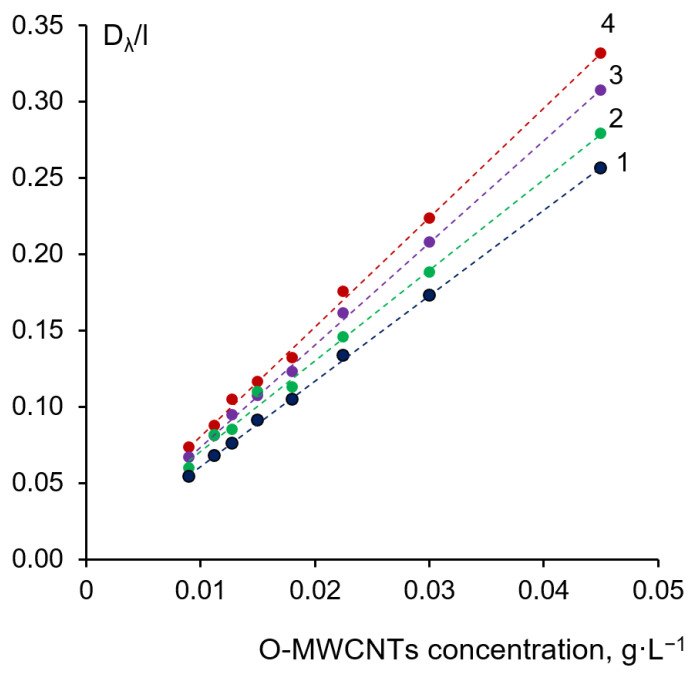
Correlation between the ratio of the absorbance to the optical path (D_λ_/l) and O-MWCNTs concentration in the dispersion at different wavelengths, nm: 1—700; 2—600; 3—500; 4—450.

To determine the actual concentration of O-MWCNT in the dispersion after the filtration, the absorption coefficient (α_λ_) was determined according to [64,65,66]. The O-MWCNTs dispersion (0.093 g·L^−1^) in NMP with the addition of 7 g·L^−1^ of PVP K-30 was prepared to plot the calibration line. The dispersion was treated by ultrasound for 180 min. The O-MWCNT dispersion with a known concentration of O-MWCNTs was diluted 2–12 times with 7 g·L^−1^ of the PVP K-30 solution in NMP to plot the calibration line (the dependence of the ratio of optical density (D_λ_) to optical path l (D_λ_/l) on O-MWCNT concentration (c)). After dilution, the solutions were treated with ultrasound for 30 min and filtered using a filter paper (Whatman, grade 1, pore size 11 μm). The absorbance intensities (D_λ_) at four different wavelengths (450, 500, 550, 700 nm) were determined using a Metertech UV/VIS SP 8001 spectrophotometer (Metertech, Taipei, Taiwan) at the wavelength range of 190–1100 nm at an increment of 1 nm. Plots of D_λ_/l (l-optical path) versus O-MWCNT concentration at different wavelengths are presented in Figure 2. The PVP K-30 spectrum was then subtracted from the spectrum of the dispersion. The slope of the plots provides the values of α_λ_ at different wavelengths. The O-MWCNT concentration was calculated for each wavelength using appropriate α_λ_ and then the average concentration was determined.

To determine the effect of dispersing the polymer (PVP K-30) concentration on the O-MWCNTs dispersion degree, the dispersions were obtained with O-MWCNTs concentration 5 g·L^−1^ and a PVP K-30 concentration of 0–50 g·L^−1^. The dispersions were treated by ultrasound for 180 min and filtered through the paper filter. The O-MWCNTs concentration after dispersion was determined according to the Equation (1):

(1) $$c=\frac{D_{\lambda }-b}{\alpha_{\lambda }}$$

where *c*—O-MWCNTs concentration after dispersion, g·L^−1^;

*D_λ_*—absorbance of the dispersion at a certain wavelength;

*b*—coefficient obtained from the equation of a straight line (*y = kx + b*) for calibration line;

α*_λ_*—absorption coefficient.

The dispersion degree (w, %) was defined as the ratio between the actual O-MWCNTs concentration (c) in dispersion (determined via the spectrophotometric technique) and the initial O-MWCNTs concentration according to Equation (2).

(2) $$w=(\frac{c}{c_{initial}})\cdot 100\%$$

#### 2.4.2. Determination of the Average Diameter and Particle Size Distribution

The average diameter (d) of particles and their particle size distribution for O-MWCNTs dispersions was determined using the dynamic light scattering technique (ZetasizerNano, Malvern Panalytical, Malvern, United Kingdom) at a scattering angle of 90° and a laser wavelength of 658.0 nm. Five parallel measurements were performed for each specimen. The experimental data processing was carried out using the software provided by the manufacturer of the instrument.

### 2.5. Preparation of Casting Solutions

The preparation of casting solutions for the fabrication of membranes was carried out using the laboratory set-up which included a mechanical overhead stirrer (IKA RW 20 Digital, IKA^®^-Werke GmbH & Co. KG, Staufen Germany), a round-bottom flask and a hot plate with glycerol bath. The solutions were stirred for 3–4 h at 120 °C and a stirring rate of 500–600 rpm. The casting solutions contained 15 wt.% PPSU, 15 wt.% PEG-20,000, 0.5 wt.% PVP K-30 and 0.03–0.19 wt.% O-MWCNT. The casting solution composition was selected according to [44], since PPSU membranes prepared from the casting solutions containing 15 wt.% PPSU, 15 wt.% PEG-20,000 in NMP demonstrated the highest pure water flux compared to membranes prepared using PEGs with molecular weights 0.4–40 kDa. The actual concentration of O-MWCNTs in the casting solution was calculated according to Equation (1). The membrane abbreviations, casting solution compositions and membrane preparation conditions are presented in Table 2.

To prepare the polymer solutions with the addition of O-MWCNTs, PPSU was first dissolved in NMP together with PEG-20,000 and PVP K-30. After the dissolution of PPSU, a calculated amount of 0.5 wt.% O-MWCNTs dispersion in 0.5 wt.% PVP K30 solution in NMP was introduced into the solution followed by stirring for 2 h. The dispersion of 0.5 wt.% O-MWCNTs in 0.5 wt.% PVP K30 solution in NMP was treated using ultrasound for 180 min and filtered through filter paper. The resulting solution was treated by ultrasound for 1 h to increase the O-MWCNTs dispersion degree.

### 2.6. Characterization of PPSU-PEG-PVP-O-MWCNT-NMP Systems

The gel point and demixing point of the PPSU-PEG-20,000-PVP K-30-O-MWCNT-NMP and PPSU-PEG-20,000-PVP K-30-NMP were determined visually after exposing 5 mL of solution in the oven for 120 min, at the preset temperature.

The dynamic viscosity of PPSU-PEG-20,000-PVP K-30-O-MWCNT-NMP and PPSU-PEG-20,000-PVP K-30-NMP systems was measured using a Brookfield DV III-Ultra (AMETEK Brookfield, Middleboro, MA, USA) rotational viscometer. The viscosity of each solution was measured under different temperatures (25 °C, 40 °C and 70 °C) at shear stress (σ) 20 N∙m^−2^.

### 2.7. Preparation of Membranes

The anisotropic flat sheet porous membranes were fabricated by means of casting the polymer solution onto the glass plate using the casting knife with a gap width of 250 µm. The codes of the membranes prepared within this study, casting solution compositions and membrane preparation conditions are shown in Table 2. To fabricate the membranes, the casting solution, the glass plate and the casting knife were preliminarily kept in the oven at 40 °C for 30 min. The glass plate with an applied layer of casting solution was immediately immersed into distilled water under different temperatures (25 °C, 40 °C and 70 °C). Thereafter, the membrane was kept in water for 24 h in order to remove residual solvent. After that, the membrane performance in ultrafiltration was studied.

### 2.8. Studies of Membrane Performance in Ultrafiltration

Pure water flux (*J_0_*) for flat sheet membranes was measured in an Amicon-type stirred filtration cell with an effective membrane area of 24.6 cm^2^ at 20 °C, transmembrane pressure of 0.1 MPa and rotational speed of 200 rpm. Before the measurements, the membranes were pressurized upon the filtration of distilled water at 0.2 MPa for 60 min. Thereafter, the membrane pure water flux studies at a transmembrane pressure of 0.1 MPa were carried out. The pure water flux was measured after 30 min of ultrafiltration. The pure water flux of membranes was defined as the volume of permeate *V* passing through unit surface area *S* per unit time *t*, (L·m^−2^·h^−1^) according to Equation (3):

(3) $$J=\frac{V}{S\cdot t}$$

where *V*—the volume of the permeate, L;

*S*—the membrane area, m^2^;

*T*—the filtration time, h.

A 0.05 wt.% HSA solution (HSA, M_n_ = 69,000 g∙mol^−1^, pI = 4.6) in 0.18 M phosphate buffer (K_2_HPO_4_–KH_2_PO_4_) with a buffer capacity of β = 0.1 and pH = 7.0 was used as the model solution for determining the membrane rejection (R, %). After the pure water flux was measured, the distilled water was replaced by HSA solution in the filtration cell. HSA solution flux and rejection were determined after 10 min of ultrafiltration according to Equations (3) and (4), respectively. Membrane rejection (*R*) was determined using Equation (4):

(4) $$R=\left(1-\frac{C_{p}}{{С}_{f}}\right)\times 100\%$$

where *C_p_* and *C_f_* are the HSA concentrations in the permeate and the feed model solution, respectively. HSA concentration in the solutions was determined from the absorbance at λ = 280 nm on a Metertech SP8001 spectrophotometer (Metertech, Taipei, Taiwan).

### 2.9. Studies of Membrane Performance in Ultrafiltration of Humic Acid (HAs) Aqueous Solution

The antifouling performance of reference PPSU and nanocomposite PPSU/O-MWCNT membranes was investigated during filtration of 0.005 wt.% HAs solution in tap water. First, pure water was filtered through studied membrane at a transmembrane pressure of 0.1 MPa for 30 min. After that, water was replaced by the HAs solution and filtered for 1 h. HAs flux (J_HAs_, L m^−2^ h^−1^), and rejection was measured every 10 min for 1 h. Thereafter, pure water was filtered for 20 min and membrane pure water flux (PWF) was determined. The flux recovery ratio (FRR, %) and total flux decline ratio (DT, %) were calculated according to Equations (5) and (6), respectively:

(5) $$\mathrm{FRR}=\left(\frac{\mathrm{JWF}}{\mathrm{PWF}}\right)\cdot 100,$$

(6) $$\mathrm{DT}=\left(\frac{\mathrm{PWF}-J_{\mathrm{HAs}}}{\mathrm{PWF}}\right)\cdot 100,$$

where JWF is water flux after HAs solution ultrafiltration, L m^−2^ h^−1^, PWF—initial pure water flux, L m^−2^ h^−1^ and J_HAs_ is flux of HAs solution, L m^−2^ h^−1^.

Feed solution characteristics were the following: color (λ = 400 nm)—0.05; total organic carbon (TOC) content—21.7 mg L^−1^; pH = 8.4.

The optical density of HAs feed solution and permeate were analyzed using a UV–vis spectrophotometer at a wavelength of 400 nm. The total organic carbon of the feed and permeate solutions was determined by a Multi N/C UV HS TOC analyzer (Analytik Jena AG, Jena, Germany).

### 2.10. Studies of Membrane Cross-Section Morphologies

The cross-section morphology of the membranes was studied using a LEO 1420 scanning electron microscope (SEM) (LEO Electron Microscopy Inc., Thornwood, NY, USA). The membrane cross-section specimens were prepared by cryogenic fracture in liquid nitrogen followed by gold sputtering in an EMITECH 550X vacuum unit. To prepare membrane samples for SEM studies, the membranes were soaked in 50 wt.% glycerol aqueous solution for 1 h and dried at ambient temperature for three days.

### 2.11. Study of the Selective Layer Surface by Atomic Force Microscopy

Topography of the membrane selective layer surface was investigated by an NT-206 atomic force microscope (AFM) (Microtestmachines, Gomel, Belarus) with standard silicon cantilevers NSC 11 A with a stiffness constant of 3 N m^−1^ (MikroMasch, Wetzlar, Germany).

The roughness R_a_ (nm) characterizes the variability along the Z surface at the scanning area of 5 × 5 μm and was estimated according to Equation (7):

(7) $$R_{a}=\frac{1}{N}\;\overset{N_{y}-1}{\underset{j=0}{\sum }}\overset{N_{x}-1}{\underset{i=0}{\sum }}\left|Z_{i,j}-\bar{Z}\right|$$

where N—number of scan matrix points; Z_i,j_—height value in position (x, y); $\bar{Z}$—arithmetic mean of the height at the whole of scanning area.

Root mean square deviation (R_q_, nm) was calculated by the Equation (8):

(8) $$R_{q}={\left(\frac{1}{N_{x}\cdot N_{y}}\;\overset{N_{y}-1}{\underset{j=0}{\sum }}\overset{N_{x}-1}{\underset{i=0}{\sum }}\left|Z_{i,j}-\bar{Z}\right|\right)}^{1/2}$$

The roughness parameters were calculated by averaging values from 10–15 microphotographs from different places of the same membrane sample.

### 2.12. Determination of Water Contact Angle

The water contact angle of the surface of the selective layer (θ,°) was determined by the attached air bubble technique using a LK-1 goniometer (“Open Science”, Krasnogorsk, Russia). The measurement error was ±2°.

## 3. Results

### 3.1. Study of Dispersions of O-MWCNT

Previously, it was found that PVP is an effective dispersing polymer for MWCNTs in N,N-dimethylacetamide (DMAc) and NMP [64,65,66]. Thus, PVP was selected as a dispersing agent in this study. Moreover, it was reported that PVP can serve as a hydrophilizing agent for PPSU for the preparation of ultrafiltration membranes [42]. To reveal the better PVP K-30 concentration for dispersing of O-MWCNTs in NMP the dispersions with initial O-MWCNTs concentration of 5.14 g·L^−1^ with the addition of 0–50 g·L^−1^ PVP K-30 were prepared and their O-MWCNT dispersion degree was determined (Table 3).

It was found that the introduction of 5 g·L^−1^ PVP K-30 into O-MWCNTs dispersions in NMP increased the dispersion degree by an average of 2.5 times compared to MWCNTs dispersion without PVP K-30 addition (Table 3). The highest O-MWCNTs dispersion degree, 76%, was achieved at a PVP K-30 concentration 5 g·L^−1^ (0.5 wt.%). With an increase in PVP K-30 concentration, the dispersion degree decreases slightly to 70–72%. It was revealed that in cases when the PVP concentration is 50 g·L^−1^, the O-MWCNTs dispersion degree decreased to 54%, which was due to the significant rise in the dispersion viscosity. An increase in viscosity prevents debundling of MWCNTs aggregates under ultrasonic treatment. Thus, the PVP K-30 concentration of 5 g·L^−1^ (0.5 wt.%) was selected for O-MWCNTs dispersion preparation as well as for PPSU casting solution preparation.

The dynamic light scattering analysis showed that the O-MWCNTs dispersion obtained with PVP K-30 concentration of 5 g·L^−1^ (0.5 wt.%) featured a narrow particle size distribution. The average particle diameter was found to be 30 nm.

### 3.2. Study of Phase State and Viscosity of Polymer Systems PPSU-PEG-PVP-O-MWCNTs-NMP

Ternary systems “polymer-non-solvent (pore former)-solvent” are often used for the preparation of porous membranes by the non-solvent induced phase separation (NIPS) method. It is known that the phase state and viscosity of the casting solution and kinetics of the phase separation of the casting solution upon contact with non-solvent determine the structure of the resulting membranes.

In this study, casting solutions contained 15 wt.% PPSU, 15 wt.% PEG-20,000, 0.5 wt.% PVP K-30 and 0.03–0.19 wt.% O-MWCNT. The concentrations of PPSU and PEG-20,000 in NMP were selected according to [44]. The PVP K-30 concentration of 0.5 wt.% was selected since it provided the maximum O-MWCNTs dispersion degree (Table 3). The threshold O-MWCNT concentration (0.19 wt.%) was chosen since large agglomerates of O-MWCNTs were observed at higher concentrations in the polymer system.

**Figure 3:**
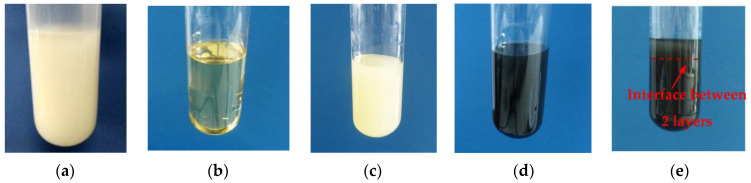
Images of 15 wt.% PPSU-15 wt.%PEG 20,000 systems in NMP at T = 20 °C (**a**) and T = 40 °C (**b**) with different additives: (**c**)—0.5 wt.% PVP K-30 at T = 40 °C; (**d**)—0.5 wt.% PVP K-30, 0.5 wt.% O-MWCNTs at T = 40 °C; (**e**)—0.5 wt.% PVP K-30, 0.5 wt.% O-MWCNTs at T = 130 °C.

The phase state and viscosity of the 15–20 wt.% PPSU-15 wt.% PEG-NMP solutions and influence of PEG molecular weight (0.4 kDa, 1 kDa, 2 kDa, 6 kDa, 20 kDa, 40 kDa) on the structure and performance of ultrafiltration membranes are reported in [6,44]. It was found previously that 15 wt.% PPSU-15 wt.% PEG-20,000–70 wt.% NMP solutions were gels at T < 37–40 °C (Figure 3a). When temperature is increased higher than the gel point (37–40 °C), the system turns into a transparent one-phase homogeneous solution which can be used for membrane casting (Figure 3b).

It was reported that the addition of 2.5–10 wt.% PVP of different molecular weights (10, 55, 360, 1300 kDa) to 20 wt.% PPSU systems in NMP yielded the formation of one phase homogeneous solution [42].

**Figure 4:**
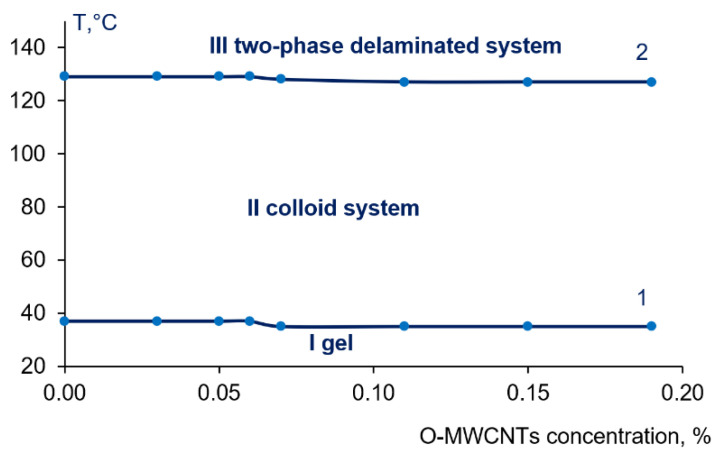
Dependence of gel point (1) and demixing temperature (2) of the system containing 15 wt.% PPSU–15 wt.% PEG 20,000–0.5 wt.% PVP K30–0-0.19 wt.% O-MWCNTs in NMP: I—gel region, II—region of colloid system, III—region of the two-phase delaminated system.

However, in this study it was found that when 0.5 wt.% PVP K-30 and O-MWCNTs additive (0.03–0.19 wt.%) were added to 15 wt.% PPSU-15 wt.% PEG-20,000-70 wt.% NMP solutions, colloid systems are formed (Figure 3c–e). A fragment of phase diagram of these systems is presented in Figure 4.

It was revealed that the PPSU–PEG-20,000–PVP K-30-NMP system and systems with an O-MWCNTs additive (0.03–0.19 wt.%) featured two critical temperatures: the temperature of the transition “solution–gel” (gel point) (T = 35–37 °C) and the demixing temperature (T = 127–129 °C) when two bulk phases are formed and the polymer system delaminates (Figure 3c–e and Figure 4). It is worth noting that the increase of O-MWCNTs concentration up to 0.07 wt.% and higher yields the decrease of gel point from 37 °C down to 35 °C and the decrease of the demixing temperature from 129 °C down to 128 °C and further down to 127 °C (Figure 4).

It is worth noting that the PPSU–PEG-20,000–PVP K-30-NMP system and systems with O-MWCNTs additive form the second-type gels according to Rogovina et al. [67]. Second-type gels are temperature-dependent heterogeneous systems with incomplete phase separation where a space network is formed due to physical interactions not due to covalent bonds. In the studied systems, the complete phase separation does not occur due to the high viscosity of the system as well as the negligible fluidity. In such systems, the gel morphology is represented by a relatively uniform distribution of submicron particles of one phase dispersed in another phase.

At temperatures above 35–37 °C, the gel transition to a state of viscous flow is observed. Compared to the 15 wt.%PPSU–15 wt.%PEG-20,000–NMP system studied previously [44], which transits to a state of the homogeneous one-phase and visually transparent solution at T = 35 ÷ 40 °C, the addition of 0.5 wt.% PVP K-30 results in the transition “gel–colloid system”.

At temperatures of 127 °C–129 °C and higher, the system is completely separated into two phases. This effect is accounted for by the association of particles belonging to the similar phase as well as phase separation by density. The lower phase has higher viscosity and is enriched by PPSU and O-MWCNTs with PVP K-30, while the upper phase (less viscous) is enriched by PEG-20,000 and PVP K-30 (Figure 3e). Concentration of MWCNTs in the phase with a higher PPSU concentration indicates the formation of strong interactions between PPSU and O-MWCNTs due to van der Waals forces, which confirms the high dispersion degree and the uniform distribution of O-MWCNTs in the PPSU solution [54].

It was found that the dynamic viscosity of 15 wt.% PPSU-15 wt.% PEG-20,000-PVP K-30 systems at 40 °C only slightly depends on the PVP K-30 concentration. It was shown that the addition of 0.5 and 2.8 wt.% of PVP K30 increases the viscosity of the solution from 2.25 up to 2.31 and 2.55 Pa·s, respectively. However, it was reported that a dramatic increase of viscosity is observed when the PVP of different molecular weights (10, 55, 360, 1300 kDa) is added to 20 wt.% PPSU systems in NMP [42]. This difference is due to the different phase states of the systems: PPSU-PVP-NMP systems are solutions; however, 15 wt.% PPSU-15 wt.% PEG-20,000-PVP K-30 was found to be a colloid system.

It was found that PPSU–PEG-20,000–PVP K-30-O-MWCNT-NMP systems at 40–60 °C feature a Newtonian flow mode within the shear stress range 8–85 N·m^−2^, as indicated by the linear correlation between shear rate and shear stress.

**Figure 5:**
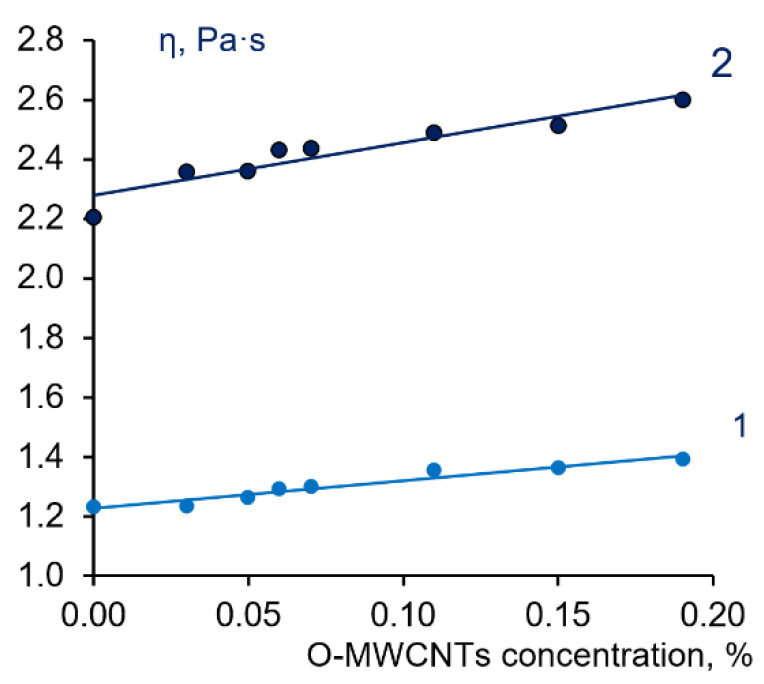
The dependence of dynamic viscosity (η) of colloid systems containing 15 wt.% PPSU–15 wt.% PEG 20,000–0.5 wt.% PVP K-30-0-0.19 wt.% O-MWCNTs in NMP on O-MWCNTs concentration at 40 °C (1) and 60 °C (2).

It was found that with an increase in O-MWCNTs concentration, the viscosity of casting solutions slightly increases both at 40 °C and 60 °C (Figure 5), which is accounted for by the formation of additional interactions between PPSU and O-MWCNTs [68].

### 3.3. Study of the Effect of O-MWCNT Addition on Membrane Structure at Different Coagulation Bath Temperatures

According to the study of the phase state and viscosity of colloid systems PPSU-PEG-20,000-PVP-O-MWCNT, a method for the preparation of highly permeable PPSU membranes is proposed. The method includes processing of the casting solution at 40 °C, since this is the temperature at which a colloid system possesses fluidity (region II at Figure 4). Thereafter, the as cast polymer film is placed into a coagulation bath (CB) at T = 25 °C which is lower than the gel point (region I at Figure 4), or at a temperature lower than the demixing temperature (40 °C and 70 °C) (region II at Figure 5). It should be noted that at a coagulation bath temperature T_cb_ = 25 °C, phase separation takes place via a combination of non-solvent induced phase separation (NIPS) and temperature induced phase separation (TIPS), while at the T_cb_ = 40 °C and T_cb_ = 70 °C only NIPS occurs. The casting solution composition and conditions of membrane preparation are listed in Table 2.

**Figure 6:**
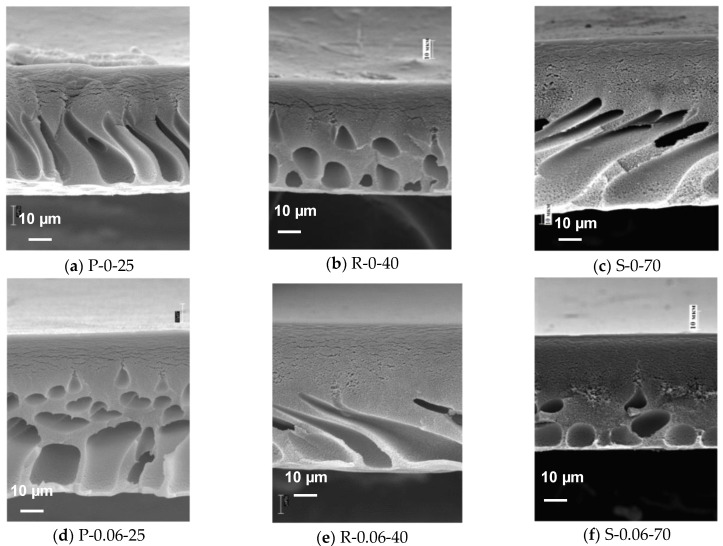

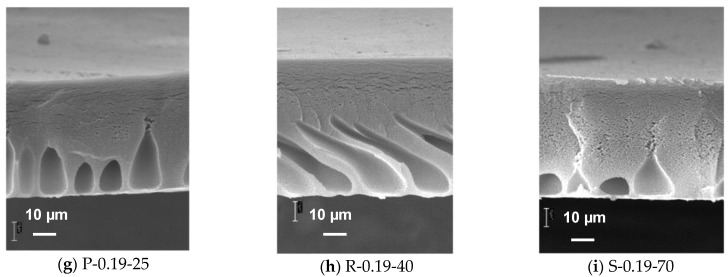
SEM micrographs of the cross-section of ultrafiltration membranes prepared from 15 wt.% PPSU–15 wt.% PEG 20,000–0.5 wt.% PVP K-30-0-0.19 wt.% O-MWCNTs in NMP at coagulation bath temperatures 25 °C (**a**–**g**), 40 °C (**b**–**h**), and 70 °C (**c**–**i**), O-MWCNTs concentrations, wt.%: (**a**–**c**)—0; (**d**–**f**)—0.06; (**g**–**i**)—0.19.

**Figure 7:**
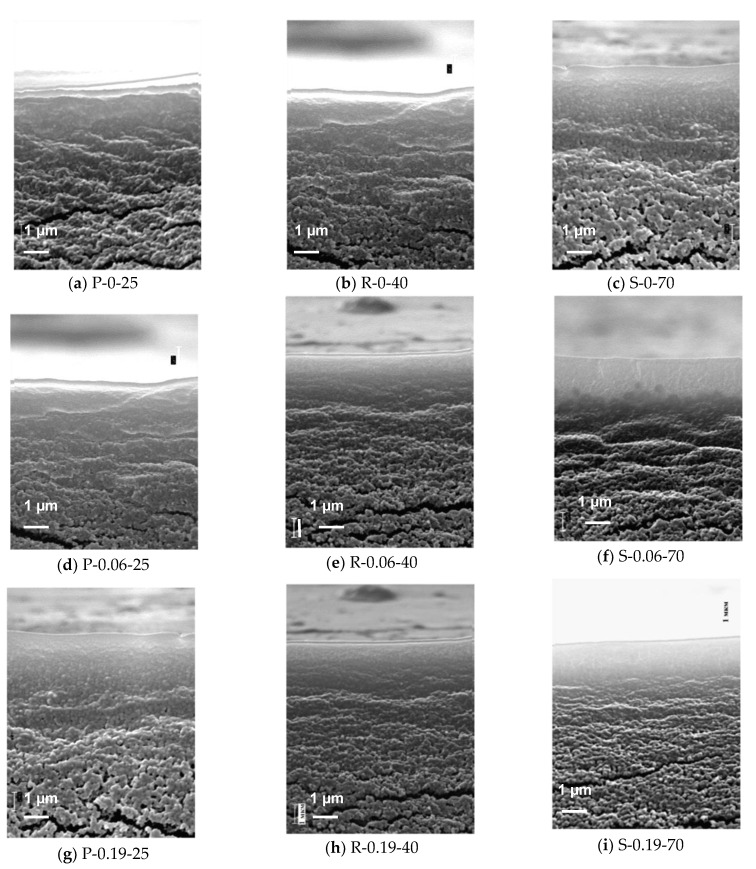
SEM micrographs of the enlarged fragment of the cross-section in the vicinity of the selective layer of PPSU and nanocomposite PPSU/O-MWCNTs ultrafiltration membranes prepared at coagulation bath temperatures 25 °C (**a**–**g**), 40 °C (**b**–**h**), and 70 °C (**c**–**i**), O-MWCNTs concentrations, wt.%: (**a**–**c**)—0; (**d**–**f**)—0.06; (**g**–**i**)—0.19.

The structures of the PPSU and PPSU/O-MWCNT membranes obtained at different coagulation bath temperatures are presented in Figure 6 and Figure 7. The cross-sections of the membranes have a highly asymmetrical structure consisting of the following layers: a thin and dense selective layer that determines membrane flux and selectivity, a transitional layer with visible structural elements; and a drainage layer playing a role of porous support and providing the mechanical strength of the membranes (Figure 6). It is worth noting that the porous structure of the selective layer cannot be captured at the given magnification.

It was found that the coagulation bath temperature significantly influences the structure of the cross-section and selective layer of the PPSU membranes (Figure 6a–c and Figure 7a–c). At a coagulation bath temperature of 25 °C, formation of elongated macrovoids is observed in the structure of the membrane drainage layer (Figure 6a). When the coagulation bath temperature increases up to 40 °C, the size of macrovoids decreases and their shape becomes round (Figure 6b). The change in the drainage layer morphology is accounted for by the different type of phase separation in the casting solution. At 25 °C, the process of phase separation which is induced by a contact with non-solvent (water) (NIPS) is associated with the phase separation resulting from temperature decrease (TIPS) (the temperature becomes lower than gel point). This coupled mechanism for PPSU solutions phase separation was firstly reported in [6,44]. At a coagulation bath temperature of 40 °C, only NIPS occurs. It is worth noting that the coagulation bath temperature of 40 °C is the most equilibrium conditions of phase separation since the temperature of the casting solution and coagulation bath are equal. Furthermore, at a coagulation bath temperature of 70 °C, the diffusion of the non-solvent into the polymer solution and of the solvent into the coagulation bath intensifies, and the resulting membrane structure is similar to that of the membranes obtained at T_cb_ = 25 °C featuring elongated macrovoids in the membrane sublayer (Figure 6a–c). Meanwhile, the membrane matrix of the drainage layer is characterized by a more porous and loose structure at T_cb_ = 70 °C compared to the membranes obtained at T_cb_ = 25 °C and T_cb_ = 40 °C (Figure 6a–c).

It was shown that the introduction of O-MWCNTs to PPSU-PEG-20,000-PVP K-30 casting solution suppresses the formation of macrovoids and shifts them down against the selective layer, particularly at the highest O-MWCNTs concentration of 0.19 wt.% at all studied coagulation bath temperatures (Figure 6). This is attributed to the increase in the viscosity of the casting solution when the O-MWCNTs concentration increases (Figure 5), which slows down the kinetics of phase separation and results in the delayed demixing mechanism of NIPS. Moreover, the structure of the dispersion is changed and additional contacts between PPSU and O-MWCNTs are formed when O-MWCNTs are added to the casting solution [54].

It was found that developed PPSU and PPSU/O-MWCNTs membranes feature relatively dense selective layer the porous structure of which cannot be detected at the magnification applied (Figure 7). The PPSU membranes at coagulant temperatures 40 °C and 70 °C have detectable globular associates in the selective layer (Figure 7b,c). With an increase in coagulant temperature, the width of the selective layer decreases and it becomes more uniform both for PPSU and PPSU/O-MWCNT membranes due to the different kinetics of phase separation. It was found that an increase in O-MWCNTs concentration in the casting solution yielded the formation of a thinner and more uniform selective layer.

The transitional layer of the membranes is a porous dense sublayer with structural elements—globular associates divided by large transverse crazes (Figure 7). The packing density of globular nodules decreases from the selective layer to the drainage layer, and the structure of the membrane matrix becomes looser. The PPSU membranes feature a layered structure of the transitional layer, whereas its drainage layer has large craze-like pores. The membranes modified by O-MWCNTs possess a higher porosity of the transitional layer compared to the reference PPSU membranes. The pores have craze-like shape and are located across the width of the transitional layer (Figure 7).

### 3.4. AFM Studies of the Selective Layer Surface of PPSU Membranes

**Figure 8:**
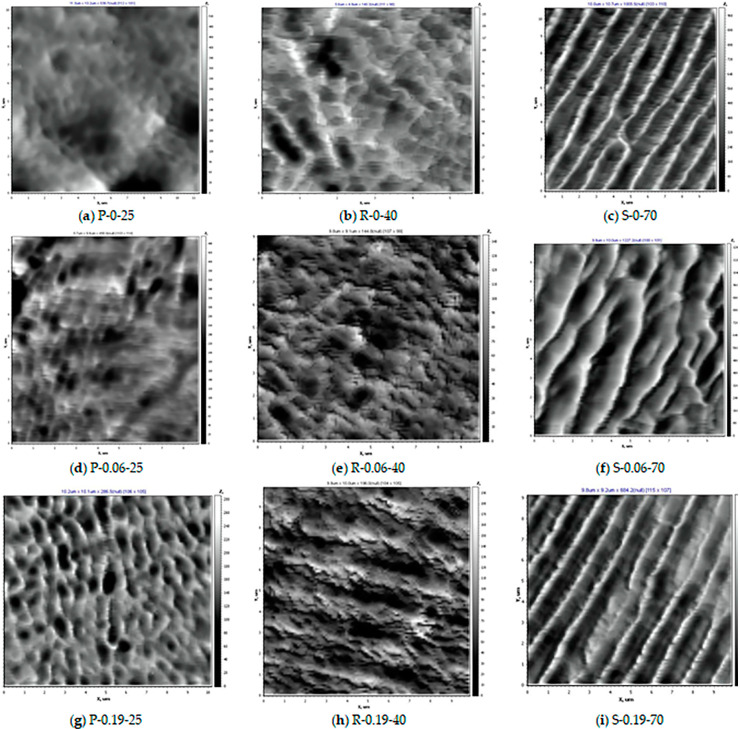
AFM images of the selective layer surfaces of of PPSU and PPSU/O-MWCNT ultrafiltration membranes prepared at coagulation bath temperatures 25 °C (**a**–**g**), 40 °C (**b**–**h**), and 70 °C (**c**–**i**), O-MWCNTs concentrations, wt.%: (**a**–**c**)—0; (**d**–**f**)—0.06; (**g**–**i**)—0.19.

The topography of the selective layer surface was studied by AFM and presented in Figure 8. Surface roughness parameters: root-mean-squared surface roughness (*R*_q_) and average roughness (*R*_a_) of PPSU and nanocomposite PPSU/O-MWCNT membranes are shown in Table 3.

It was found that coagulation bath temperature has a significant impact on the topography of the surface of the selective layer both for PPSU and nanocomposite PPSU/O-MWCNT membranes.

It was found that the selective layer surface of the P-0-25 membrane prepared at T_cb_ = 25 °C featured slightly pronounced nodular morphology and is rather smooth, which is confirmed by the low values of surface roughness parameters (Figure 8a, Table 4). The increase in the coagulation bath temperature up to 40 °C yields the formation of oval and round-shaped cavities with well-defined edges (Figure 8b).

However, the surface roughness parameters are practically not changed when coagulation bath temperatures increase up to 40 °C (Table 4). The difference of the topology of the selective layer surface is due to the difference of the mechanism and kinetics of phase separation at T_cb_ = 25 °C and T_cb_ = 40 °C. When the coagulation bath temperature is 25 °C the phase separation upon the contact with coagulant (water) is accompanied by the gel formation due to temperature decrease lower than the gel point. When as cast polymer film is immersed in the coagulation bath with T_cb_ = 25 °C, the vitrification of the casting solution occurs due to gel formation and then the coagulant diffuses into the formed gel-like medium. It leads to the formation of a smoother surface of the selective layer as the exchange rate of the coagulant diffusing into the as cast polymer film and solvent diffusing out the polymer film is not very high (Figure 8a). When T_cb_ = 40 °C, phase separation occurs only due to the NIPS mechanism and the solvent-non-solvent exchange rate becomes faster, resulting in the formation of round-shaped nodules with well-defined ridges (Figure 8b). When the coagulation bath temperature increases up to 70 °C, the highly-ordered surface topology is formed, consisting of elongated structural elements with high sharp edges (Figure 8c) which yield a substantial increase in surface roughness parameters (Table 4). The formation of such a topology pattern is difficult to explain, however, the reason for increasing the surface roughness parameters may be attributed to the increase in the solvent-non-solvent exchange rate in NIPS when the coagulation bath temperature is increased.

It was found that an increase in the O-MWCNTs concentration in the casting solution yielded the increase of surface roughness parameters at all studied coagulation bath temperatures due to the presence of O-MWCNT agglomerates (Table 4). When O-MWCNTs are added to the casting solution, the topology of the P-0.06-25 and P-0.19-25 membranes prepared at T_cb_ = 25 °C changes: the size of oval and round cavities increases and their ridges become sharper (Figure 8d,g), which leads to the gradual increase of surface roughness parameters (Table 4). This is due to the presence of O-MWCNT agglomerates in the casting solution. However, the topology of the membrane selective layer surface of R-0.06-40 and R-0.19-40 prepared at a coagulation bath temperature 40 °C differs from the topology of the P-0.06-25 and P-0.19-25 membranes prepared at T_cb_ = 25 °C due to the difference of phase separation mechanism (Figure 8d,e,g,h). For the R-0.06-40 and R-0.19-40 membranes, agglomerates of O-MWCNT appear on the membrane surface. However, an increase in surface roughness parameters is not so high compared to the reference R-0-40 membrane (Table 4). The highest increase in surface roughness parameters is observed for nanocomposite PPSU/O-MWCNT membranes obtained at a coagulation bath temperature of 70 °C (S-0.06-70 and S-0.19-70) (Figure 8f,i). It was found that the surface topology of nanocomposite PPSU/O-MWCNT membranes prepared at T_cb_ = 70 °C featured a similar pattern as the reference S-0-70 membrane with elongated structural elements with sharp ridges. However, for the S-0.06-70 membrane the elongated cavities are wider, which may be attributed to more equilibrium conditions of phase separation (Figure 8f).

### 3.5. Effect of O-MWCNTs Content on Water Contact Angle of Membrane Selective Layer

**Figure 9:**
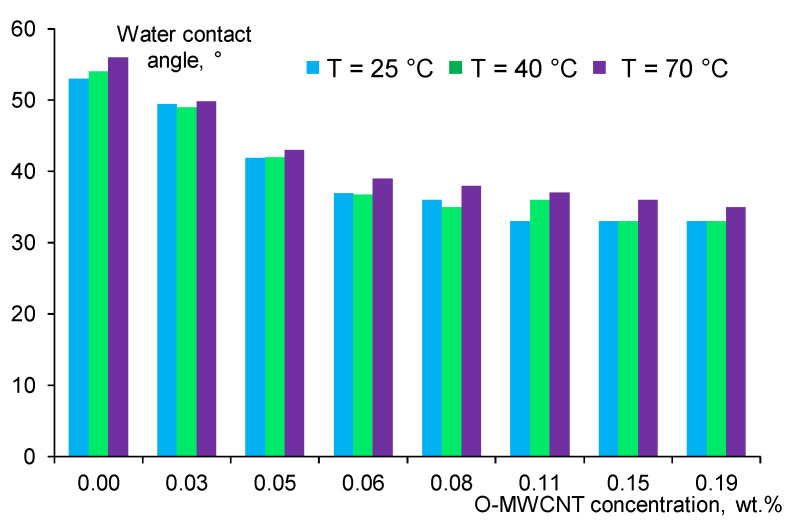
Effect of O-MWCNTs concentration in the casting solution on the water contact angle of the PPSU and nanocomposite PPSU/O-MWCNT membranes prepared at different coagulation bath temperatures: blue color—T_cb_ = 25 °C; green color—T_cb_ = 40 °C and violet color—T_cb_ = 70 °C.

The hydrophilic-hydrophobic balance of the membrane selective layer surface is usually described in terms of water contact angle values. The contact angle values were measured for the PPSU and nanocomposite PPSU/O-MWCNT membranes obtained at different coagulation bath temperatures. The dependence of the water contact angle on the O-MWCNTs content in the casting solution is presented in Figure 9.

It is worth noting that the water contact angle values of the reference PPSU membranes (P-0-25, R-0-40 and S-0-70) without O-MWCNTs additive prepared at different coagulation bath temperatures are significantly lower (53–56°) than that of the pure PPSU membranes reported in the literature (85.5–93.4°) [45]. This effect is accounted for by the PVP K-30 presence in the casting solution. PVP K-30 used to disperse O-MWCNTs increases hydrophilicity of the PPSU membrane surface of the selective layer, which is consistent with the results reported in [42]. It was found that an increase in coagulation bath temperature increased the water contact angle, especially at T_cb_ = 70 °C, due to the increase in surface roughness (Figure 8, Table 4). It is widely known that the contact angle is influenced not only by the chemical nature of the surface but also by surface topography [69]. This trend is observed both for the reference PPSU and the nanocomposite PPSU/O-MWCNT membranes (Figure 8).

It was revealed that the addition of O-MWCNTs and the increase in their concentration in the casting solution led to the substantial hydrophilization of the surface of the membrane selective layer due to the presence of hydrophilic oxygen-containing groups (hydroxyl, carbonyl, carboxylic, anhydride groups). A decrease in the water contact angle of the selective layer surface was observed as a result of the modification of PPSU membranes by hydrophilic nanoparticles and reported in [2,25,30,36,48,51,54]. The minimum value of the water contact angle 33 ± 2° was achieved for P-0.11-25, P-0.15-25, P-0.19-25, R-0.15-40 and P-0.19-40 nanocomposite membranes (Figure 8). Similar to the trend observed for reference PPSU membranes, an increase in coagulation bath temperature leads to a slight increase in water contact angle (1–4°) due to the increase in surface roughness of the membrane selective layer (Figure 8, Table 4). The difference between contact angle values for different coagulation bath temperatures is also due to the migration of hydrophilic O-MWCNTs to the membrane surface during the phase inversion process and their different localization on the selective layer surface [54].

### 3.6. Study of the PPSU and Nanocomposite PPSU/O-MWCNT Membranes Separation Performance

**Figure 10:**
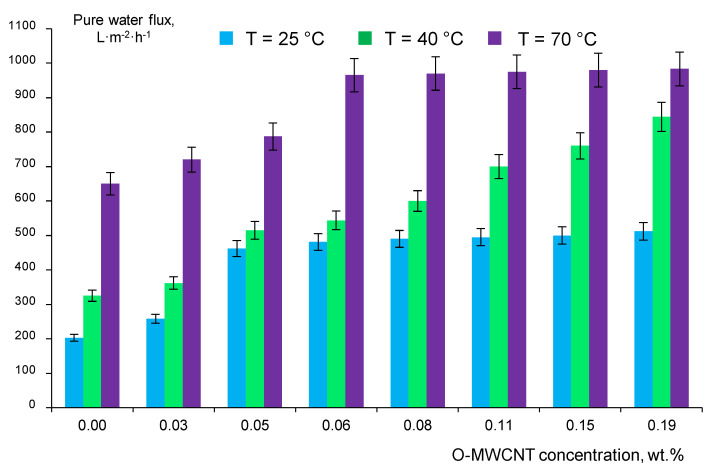
Pure water flux versus O-MWCNTs concentration in the casting solution, coagulation bath temperature: blue color—T_cb_ = 25 °C; green color—T_cb_ = 40 °C and violet color—T_cb_ = 70 °C.

The performance of PPSU and nanocomposite PPSU/O-MWCNT membranes in ultrafiltration was studied using distilled water and 0.05 wt.% human serum albumin (HSA) buffered solution (pH = 7.0) as feeds. The effect of O-MWCNTs concentration in the casting solution on the membrane pure water flux is depicted in Figure 10.

It can be seen from Figure 10 that reference P-0-25, P-0-40 and P-0-70 membranes demonstrate relatively high pure water flux (203, 325 and 650 L·m^−2^·h^−1^) compared to other PPSU ultrafiltration membranes reported in the literature (Table 1). Pure water flux was found to increase with the increase in coagulation bath temperature due to the formation of a thinner and more uniform selective layer which is in good accordance with the results reported in [6,44]. The increase in coagulation bath temperature up to 70 °C was revealed to yield the highest increase in pure water flux both for PPSU and PPSU/O-MWCNT membranes. The increase in O-MWCNT concentration in the casting solution yields a significant increase in membrane permeability due to the formation of a thinner selective layer as well as the increase in hydrophilicity of the membrane surface (Figure 7, Figure 9 and Figure 10). The highest pure water flux demonstrates S-0.19-70 membrane at all studied coagulation bath temperatures (512 L m^−2^ h^−1^ at T_cb_ = 25 °C; 844 L m^−2^ h^−1^ at T_cb_ = 40 °C and 983 L m^−2^ h^−1^ at T_cb_ = 70 °C) (Figure 10).

**Figure 11:**
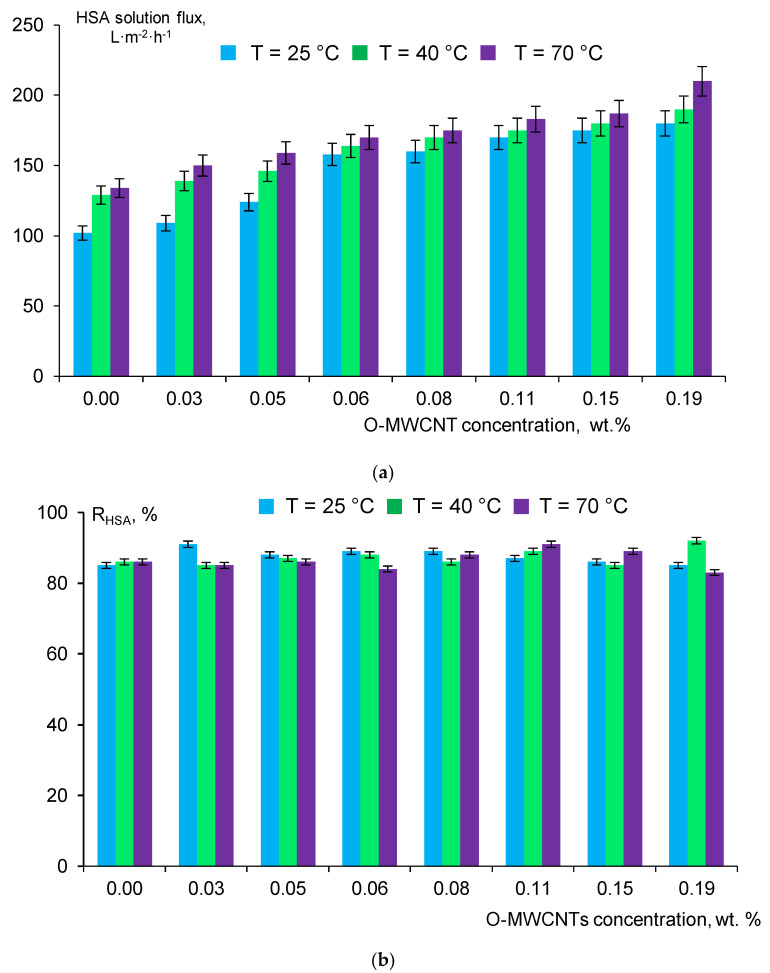
Dependence of 0.05 wt.% HSA solution flux (**a**) and HSA rejection (**b**) on O-MWCNTs concentration in the casting solution, coagulation bath temperature: blue color—T_cb_ = 25 °C; green color—T_cb_ = 40 °C and violet color—T_cb_ = 70 °C.

Similar correlations between coagulation bath temperatures, O-MWCNT concentration in the casting solution and the HSA solution flux of the membranes were revealed and presented in Figure 11.

The highest HSA solution flux was achieved for the S-0.19-70 membrane at all studied coagulation bath temperatures (180 L m^−2^ h^−1^ at T_cb_ = 25 °C; 190 L m^−2^ h^−1^ at T_cb_ = 40 °C and 210 L m^−2^ h^−1^ at T_cb_ = 70 °C) (Figure 11). It is worth noting that HSA rejection values are almost equal (83–92%) for the reference PPSU and nanocomposite PPSU/O-MWCNT membranes regardless O-MWCNT concentration in the casting solution and coagulation bath temperature (Figure 11). The lowest HSA rejection of 84% and 83% featured S-0.06-70 and S-0.19-70 membranes.

Thus, the modification of PPSU membranes by the incorporation of O-MWCNTs into the casting solution yields a substantial increase in the membrane pure water and HSA solution flux preserving high levels of HSA rejection. According to the literature analysis presented in Table 1 it can be concluded that the developed PPSU/O-MWCNT membranes demonstrate extremely high pure water flux which significantly surpasses the performance of PPSU ultrafiltration membranes reported to date, while maintaining a high level of human serum albumin rejection. Moreover, the developed PPSU/O-MWCNT are characterized by very a low water contact angle of the selective layer (35–33°) compared to the PPSU membranes reported recently (Table 1).

### 3.7. Study of Antifouling Perfromance during Humic Acid Solution Ultrafiltration

To study the influence of O-MWCNT introduction into the casting solution on membrane antifouling performance, the experiments on ultrafiltration of 0.005 wt.% HAs solution in tap water were carried out using the reference R-0-40 and nanocomposite R-0.06-40 membranes. The choice of these membranes is attributed to their moderate pure water flux and the surface roughness of the membrane selective layer (Figure 8, Figure 10 and Figure 11a, Table 4). It is widely known that antifouling performance in ultrafiltration is determined by the pore size, surface roughness, hydrophilicity and zeta potential of the membrane selective layer [70,71,72,73].

Membrane performance and antifouling parameters (flux recovery ratio (FRR) and total flux decline ratio (DT)), as well as permeate characteristics, are presented in Table 5. It was revealed that the modification of the PPSU membrane by introduction of 0.06 wt.% O-MWCNTs into the casting solution results in the decrease of the HAs solution flux from 160 to 145 L m^−2^ h^−1^ but increases the HAs rejection from 73% to 83% (Table 5). The nanocomposite R-0.06-40 membrane demonstrated better antifouling performance compared to the R-0-40 membrane: higher FRR and lower DT (Table 5). Moreover, R-0.06-40 was more effective in total organic carbon removal compared to the reference membrane (Table 5). The better antifouling performance of the nanocomposite membranes is due to the significantly higher hydrophilicity of the selective layer surface (the water contact angle is 37 ± 2°for nanocomposite R-0.06-40 versus 54 ± 2°for the reference R-0-40) at the practically similar surface roughness parameters (Table 4). The results obtained are consistent with recent studies [2,3,25,30,31,45,54]. The improvement of membrane antifouling stability as a result of the blending of the PPSU casting solution with hydrophilic nanoparticles was reported in [2,3,25,30,31,45,54].

It can be concluded that the nanocomposite PPSU/O/MWCNT membrane was more effective in HAs solution ultrafiltration compared to the reference PPSU membrane.

## 4. Conclusions

The effect of O-MWCNT loading to the PPSU-polyethylene glycol (PEG-20,000, M_n_ = 20,000 g·mol^−1^)-polyvinylpyrrolidone (PVP K-30, M_n_ = 40,000 g·mol^−1^)-N-methy-2-pyrrolidinone (NMP) colloid systems on the phase state and viscosity was studied. It was found that the PPSU-PEG-20,000-PVP K-30-O-MWCNT-NMP colloid systems feature a gel point (T = 35–37 °C) and demixing temperature (T = 127–129 °C) at which two bulk phases are formed and the polymer system delaminates. According to the study of the phase state and viscosity of these colloid systems, a method for preparation of high flux PPSU membranes is proposed which includes processing of the casting solution at a temperature higher than gel point (40 °C), and using a coagulation bath temperature lower than the gel point (25 °C) or lower than the demixing temperature (40 °C and 70 °C). It was found that the modification of PPSU ultrafiltration membranes by O-MWCNTs yielded the hydrophilization of the membrane surface and the formation of a thinner selective layer which results in the increase in membrane flux while maintaining a high level of human serum albumin rejection. It was revealed that the nanocomposite membrane demonstrated better antifouling performance and higher total organic carbon removal compared to the reference PPSU membrane in the HAs solution ultrafiltration. The developed nanocomposite PPSU/O-MWCNT membranes can be effective for water treatment and purification. This study demonstrates that the combination of two modification approaches, the blending the PPSU casting solution with hydrophilic O-MWCNT and the application of the solutions with critical solution temperatures, allows for the obtaining of highly hydrophilic PPSU ultrafiltration membranes with an extremely high pure water flux which surpasses the permeability of the PPSU membranes reported to date. The results obtained within this work provide a platform for future studies on the development of highly permeable and hydrophilic PPSU membranes, which will broaden the scope of their application.

## Figures and Tables

**Table 1:** Performance of PPSU ultrafiltration membranes reported in the literature to date.

Casting Solution	Nanofiller		Performance		Water Contact Angle, °	FRR ^2^, %	Reference
	Type	Content, wt.%	Pure Water Flux, L·m^−2^·h^−1^	Rejection of BSA ^1^, %			
17 wt.% PPSU in NMP	-	-	177 at 0.3 MPa	-	-	-	[36]
	GO	0.05–0.4	174–211 at 0.3 MPa	-	46–60	-	
PPSU:PVP ^3^:Tween-80:PG ^4^:NMP = 15:9:4.5:1.5:70	-	-	183.4 at 0.1 MPa	70.1	54.4	-	[43]
PPSU:PVP:Tween-80:PG:NMP = 15:6:3:1:75	-	-	148 at 0.1 MPa	81	63	-	
22 wt.% PPSU-5 wt.% PEG-20,000 in NMP	-	-	183 at. 0.5 MPa	100	61.9	63.2	[38]
14 wt.% PPSU in NMP	-	-	37.69 at 0.1 MPa	52.69	49.38	-	[53]
18 wt.% PPSU-2 wt.% PVP ^5^ in NMP	Zeolite ZSM-5	0.4	113.98 at 0.3 MPa	100	69	68.78	[31]
22 wt.% PPSU-3 wt.% PVP ^6^ in DMAc	-	-	140 at 0.28 MPa	99.9	68.2	85	[40]
16 wt.% PPSU-4 wt.% PVP in NMP	BiOCl on activated charcoal	2	465 at 0.2 MPa	-	~67	53.33	[30]
25 wt.% PPSU	TiO_2_	0.5	53 at 0.2 MPa	95	46	60	[2]
15 wt.% PPSU-6 wt.% PEG-6000 in DMAc	GO	1.5	~220 at 0.1 MPa	94	65	86	[45]
20% wt.% PPSU-15 wt.% PEG-6000 in NMP	-	-	486 at 0.1 MPa	89 (HSA)	76	-	[6]
15 wt.% PPSU in NMP	CuO/g-C_3_N_4_	0.5	202 at 0.069 MPa	96	53	79	[25]
16 wt.% PPSU-4 wt.% PVP ^5^ in NMP	SnO_2_	0.4	362.9 at 0.2 MPa	-	63.77	-	[3]
15 wt.% PPSU in NMP	Gum arabic-graphene	0.15	119 at 0.4 MPa	88 (sodium alginate)	50	-	[51]
35 wt.%PPSU- 5 wt.%PEI-6 wt.% PEG	Activated carbon	0.25	184 at 0.1 MPa	80 (humic acids)	116.6	-	[50]
10 wt.% PPSU-10 wt.% PES in NMP	SiO_2_	2.5	57 at 0.5 MPa	80.49	52.57	-	[48]
17.5 wt.% PPSU in NMP	MWCNT	0.5	46.6 at 0.0345 MPa	~97	65	-	[54]
	O-MWCNT ^8^	0.5	56.7 at 0.0345 MPa	~95	45	-	
15 wt.% PPSU-15 wt.% PEG-20,000- 0.5 wt.% PVP ^7^ NMP	O-MWCNT	0.06	544 at 0.1 MPa	88 (HSA) ^9^ 83 (humic acids)	35	62 (humic acids)	This study
		0.19	983 at 0.1 MPa	83 (HSA) ^9^	35		

^1^ Bovine serum albumin unless otherwise specified. ^2^ Flux recovery ratio (FRR) after ultrafiltration of BSA solution unless otherwise specified. ^3^ M_w_(PVP) = 15,000 g·mol^−1^. ^4^ 1,2-propylene glycol. ^5^ M_w_(PVP) = 360,000 g·mol^−1^. ^6^ M_w_(PVP) = 10,000 g·mol^−1^. ^7^ M_w_(PVP) = 40,000 g·mol^−1^. ^8^ carboxylated MWCNT. ^9^ human serum albumin.

**Table 2:** Abbreviations of membranes, casting solution compositions and conditions of membrane preparation.

Membrane Code	Casting Solution Composition, wt.%					Preparation Conditions	
	PPSU	PEG-20,000	PVP K-30	O-MWCNT	NMP	Temperature of Casting Solution, °C	Temperature of Coagulation Bath, °C
P-0-25	15	15	0.5	0	69.50	40	25
P-0.03-25	15	15	0.5	0.03	69.47	40	25
P-0.05-25	15	15	0.5	0.05	69.45	40	25
P-0.06-25	15	15	0.5	0.06	69.44	40	25
P-0.07-25	15	15	0.5	0.07	69.43	40	25
P-0.11-25	15	15	0.5	0.11	69.39	40	25
P-0.15-25	15	15	0.5	0.15	69.35	40	25
P-0.19-25	15	15	0.5	0.19	69.31	40	25
R-0-40	15	15	0.5	0	69.50	40	40
R-0.03-40	15	15	0.5	0.03	69.47	40	40
R-0.05-40	15	15	0.5	0.05	69.45	40	40
R-0.06-40	15	15	0.5	0.06	69.44	40	40
R-0.07-40	15	15	0.5	0.07	69.43	40	40
R-0.11-40	15	15	0.5	0.11	69.39	40	40
R-0.15-40	15	15	0.5	0.15	69.35	40	40
R-0.19-40	15	15	0.5	0.19	69.31	40	40
S-0-70	15	15	0.5	0	69.50	40	70
S-0.03-70	15	15	0.5	0.03	69.47	40	70
S-0.05-70	15	15	0.5	0.05	69.45	40	70
S-0.06-70	15	15	0.5	0.06	69.44	40	70
S-0.07-70	15	15	0.5	0.07	69.43	40	70
S-0.11-70	15	15	0.5	0.11	69.39	40	70
S-0.15-70	15	15	0.5	0.15	69.35	40	70
S-0.19-70	15	15	0.5	0.19	69.31	40	70

**Table 3:** O-MWCNTs dispersion degree (w) depending on the PVP K-30 concentration.

PVP K-30 Concentration, g·L^−1^	w, %	Actual Concentration of O-MWCNTs in the Dispersion, g·L^−1^
0.0	30	1.5
5.0	76	3.9
7.0	72	3.7
15.0	71	3.7
20.0	71	3.7
30.0	70	3.6
50.0	54	2.8

**Table 4:** Surface roughness parameters of the selective layer surface of PPSU and nanocomposite PPSU/O-MWCNT ultrafiltration membranes prepared at different coagulation bath temperatures.

Membrane Abbreviation	Roughness Parameters	
	R_a_ [nm]	R_q_ [nm]
P-0-25	2.9	3.8
P-0.06-25	5.0	6.4
P-0.19-25	5.4	6.9
R-0-40	3.0	3.8
R-0.06-40	3.7	4.9
R-0.19-40	5.1	6.3
S-0-70	6.0	8.1
S-0.06-70	18.1	22.4
S-0.19-70	19.7	24.3

**Table 5:** HAs solution flux, antifouling parameters and permeate characteristics of reference and modified membranes.

Membrane Abbreviation	J_HAs_ [L m^−2^ h^−1^]	R, %	FRR [%]	DT [%]	Permeate Parameters		
					Color (λ = 400 nm)	pH	TOC [mg L^−1^]
R-0-40	160	73	47	45	0	8.4	5.57
R-0.06-40	145	83	62	37	0	8.3	3.66

## Data Availability

The data presented in this study are available on request from the corresponding author.
